# A Positive Trajectory for Corals at Little Cayman Island

**DOI:** 10.1371/journal.pone.0075432

**Published:** 2013-10-09

**Authors:** Carrie Manfrino, Charles A. Jacoby, Emma Camp, Thomas K. Frazer

**Affiliations:** 1 Earth, Atmosphere, and Ocean Sciences, Kean University, Union, New Jersey, United States of America; 2 Central Caribbean Marine Institute, Princeton, New Jersey, United States of America; 3 Soil and Water Science Department, University of Florida, Gainesville, Florida, United States of America; 4 Central Caribbean Marine Institute, Little Cayman Research Centre, Little Cayman, Cayman Islands; 5 School of Natural Resources and Environment, University of Florida, Gainesville, Florida, United States of America; 6 Fisheries and Aquatic Sciences Program, School of Forest Resources and Conservation, University of Florida, Gainesville, Florida, United States of America; University of Connecticut, United States of America

## Abstract

Coral reefs are damaged by natural disturbances and local and global anthropogenic stresses. As stresses intensify, so do debates about whether reefs will recover after significant damage. True headway in this debate requires documented temporal trajectories for coral assemblages subjected to various combinations of stresses; therefore, we report relevant changes in coral assemblages at Little Cayman Island. Between 1999 and 2012, spatiotemporal patterns in cover, densities of juveniles and size structure of assemblages were documented inside and outside marine protected areas using transects, quadrats and measurements of maximum diameters. Over five years, bleaching and disease caused live cover to decrease from 26% to 14%, with full recovery seven years later. Juvenile densities varied, reaching a maximum in 2010. Both patterns were consistent within and outside protected areas. In addition, dominant coral species persisted within and outside protected areas although their size frequency distributions varied temporally and spatially. The health of the coral assemblage and the similarity of responses across levels of protection suggested that negligible anthropogenic disturbance at the local scale was a key factor underlying the observed resilience.

## Introduction

Coral reefs occupy less than 0.01% of the marine environment, yet they harbor up to 25% of marine biodiversity, yield approximately 25% of the fish catch in developing nations, and generate up to 30% of export earnings in 100 countries that promote reef-related tourism [1]. These benefits may disappear because coral reefs around the world are being degraded by local and global anthropogenic stressors that cause damage beyond that due to natural disturbances, such as large storms, hurricanes, exposure to unusually low tides, and freshwater inputs [2]. Local anthropogenic stressors, including sediment loads, organic and inorganic pollution, physical damage, and overfishing, have their negative effects on corals exacerbated by stresses arising from global anthropogenic changes, such as bleaching in response to warmer sea surface temperatures, reduced calcification due to ocean acidification, and more frequent damage from storms as weather patterns become more extreme [1]–[6]. In fact, local stressors threaten more than 60% of reefs, and threats expand to approximately 75% of reefs with the additional consideration of coral bleaching due to thermal stress [1].

Such statistics have engendered debates about the sustainable management of coral reefs, including the value of marine protected areas with or without no-take zones [7], [8]. Managed areas of any type, even those with stringent enforcement, provide no direct protection from natural cataclysms and global perturbations [4], [7], [9], [10]. Nevertheless, managed areas of sufficient size and connectivity may promote resilience through bottom-up effects, e.g., by providing refuges for reproducing corals and enhancing regional recruitment through larval exchange [4]. Moreover, protection from overfishing in no-take zones and protected areas that connect multiple habitats may generate beneficial top-down effects by fostering robust fish and invertebrate assemblages that include herbivores grazing on algae that could otherwise usurp the open space needed by coral larvae or regenerating fragments [4], [11]. Regardless of the mechanism, understanding the conditions that promote recovery of damaged coral reefs remains critical to the formulation of effective management actions.

Studies documenting recovery of corals have yielded mixed conclusions. Fine-scale surveys and experimental studies indicate diverse responses or the lack of a short-term response, which may be due to i) local oceanographic, meteorological and ecological conditions that ameliorate or exacerbate stress from disturbances; ii) variable growth and regeneration rates among coral taxa; iii) variation in larval supply; iv) differences in type and extent of the most recent disturbance; and v) unique interactions among coincident and sequential disturbances [12]–[21]. Data analyzed in broad-scale meta-analyses also suffer from the effects of these influences, along with biases from the non-random placement of managed areas and significant variation in enforcement [22]–[24]. Although two meta-analyses report little or slow recovery of corals in managed areas [22], [23], one study describes increased coral cover within protected areas and decreased cover on unprotected reefs [24]. In contrast, a recent study ascribes recovery from major disturbance to minimal local anthropogenic stresses and long distance recruitment rather than the presence of a protected area [25]. Given these inconsistencies, there remains a need for long-term studies employing sampling designs that document conditions within and outside of managed areas [14], [23], [24]. Eventually, a synthesis of relevant results will deliver insights required for sustainable management of coral reefs and the values they deliver.

As a contribution to this goal, this long-term study examines temporal trajectories for coral reefs in protected and unprotected areas off Little Cayman Island. All data were derived from nondestructive observations that were in full compliance with conditions set by the Cayman Islands Marine Conservation Board. Data collected during 10 of the last 14 years provide the basis for testing null hypotheses related to the interactions between marine protected areas and the effects of bleaching and disease, i.e., bleaching and disease led to no significant temporal changes or spatial differences in i) cover of live coral, ii) abundance of juvenile corals, and iii) taxonomic composition and size structure of live coral assemblages within and outside of marine protected areas.

## Materials and Methods

**Figure 1:**
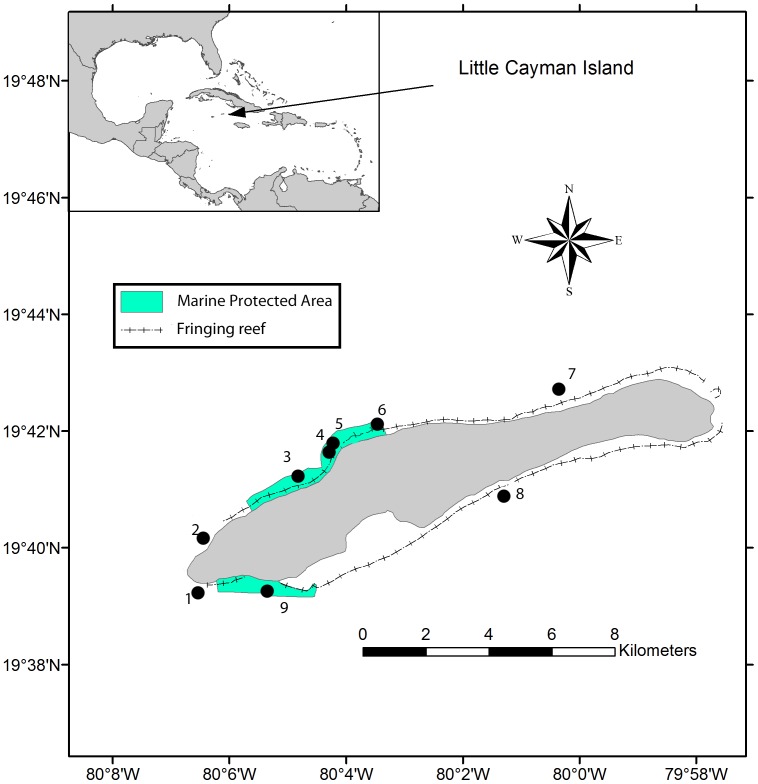
Maps showing the Caribbean region (inset) and Little Cayman Island. Marine Protected Areas shaded green and buoys used in sampling indicated by •. Coordinates for buoys: 1 = 19°39′18″N, 80°01′24″W; 2 = 19°40′17″N, 80°06′12″W; 3 = 19°41′06″N, 80°04′41″W; 4 = 19°41′33″N, 80°04′07″W; 5 = 19°41′40″N, 80°04′10″W; 6 = 19°42′03″N, 80°03′25″W; 7 = 19°42′25″N, 80°00′44″W; 8 = 19°40′51″N, 80°01′24″W; 9 = 19°39′26″N, 80°05′22″W.

Little Cayman, Cayman Islands is a 17 × 2 km, flat, low-lying island that lies 120 km northeast of Grand Cayman, 145 km south of Cuba and 10 km southwest of Cayman Brac (Fig. 1). Live coral cover around the island has been documented to be equivalent to or higher than other locations in the Caribbean [26]. Two key factors contribute to this status, i.e., the lack of freshwater rivers on the island and minimal anthropogenic stress due to nutrient inputs, commercial fishing, groundings or other activities undertaken by <200 permanent residents. Since the mid-1980s, marine protected areas have been enforced along more than 50% of the island’s coast, including two no-take marine parks. Despite protection and limited anthropogenic pressure, reefs surrounding Little Cayman experienced a decline of live coral cover from 26% to 14% between 1999 and 2004 [9]. This five-year decline was attributed primarily to white plague, which began after extended thermal stress caused significant bleaching in 1998 [9], [27], [28]. In addition, up to 22% of corals off Little Cayman Island exhibited bleaching in 2003 and 2005 in response to elevated temperatures throughout the Caribbean [27], and >5% of *Agaricia* spp., *Diploria* spp., *Montastraea* spp., *Porites* spp. or *Siderastrea* spp. showed signs of bleaching in 2009 [28].

In July and early August, shallow (9–13 m) spur and groove reefs around Little Cayman were surveyed in 1999, annually from 2002 to 2007, and annually from 2009 to 2012 [9]. Surveys were based on the Atlantic and Gulf Rapid Reef Assessment techniques that are designed to document broad-scale and long-term status and trends [29]. Surveys employed haphazard, 10-m line intercept transects along spurs. To ensure dispersion and more rigorous representation of island-wide conditions, transects were distributed around multiple, permanent mooring buoys on either the leeward or windward side of the island. Across all years of sampling, coral assemblages were surveyed along 8–37 transects inside marine protected areas and 18–61 transects outside marine protected areas as dictated by weather and logistics.

Cover of live corals was analyzed for 9 years when ≥10 transects were surveyed at ≥2 mooring buoys both inside and outside marine protected areas (Table 1). The distances beneath each transect line occupied by live coral were measured to the nearest 1 cm. The total distance along each replicate transect occupied by live coral was converted to proportional cover for the transect by dividing it by the total length of the transect minus the distance occupied by sandy substrate unsuitable for corals.

**Table 1:** Distribution of samples for key parameters. – = category not relevant.

Parameter	Year	Protected				Not protected			
		Buoys	Transects	Quadrats	Colonies	Buoys	Transects	Quadrats	Colonies
Live coral cover	1999	2	22	–	–	3	39	–	–
	2002	2	21	–	–	3	29	–	–
	2004	2	18	–	–	3	29	–	–
	2006	2	17	–	–	3	24	–	–
	2007	2	22	–	–	3	30	–	–
	2009	2	13	–	–	3	21	–	–
	2010	2	22	–	–	3	30	–	–
	2011	2	11	–	–	3	18	–	–
	2012	2	12	–	–	3	18	–	–
Juvenile density	1999	3	–	185	–	5	–	300	–
	2002	2	–	95	–	3	–	140	–
	2004	3	–	135	–	5	–	250	–
	2005	2	–	105	–	2	–	130	–
	2006	3	–	125	–	5	–	190	–
	2007	2	–	110	–	5	–	235	–
	2009	3	–	100	–	5	–	155	–
	2010	3	–	90	–	3	–	90	–
	2011	3	–	85	–	5	–	150	–
	2012	5	–	150	–	5	–	150	–
Coral diameter	1999	3	–	–	113	5	–	–	174
	2002	2	–	–	79	3	–	–	131
	2004	3	–	–	131	5	–	–	177
	2005	2	–	–	62	2	–	–	72
	2006	3	–	–	104	5	–	–	177
	2007	2	–	–	56	5	–	–	169
	2009	3	–	–	82	5	–	–	92
	2010	3	–	–	78	3	–	–	81
	2011	3	–	–	94	5	–	–	114
	2012	4	–	–	96	5	–	–	97

Coral recruitment was estimated by counting colonies that were ≤2 cm in diameter. These colonies were counted in 625-cm^2^ quadrats placed every 2 m along the 10-m transects. For 10 years when ≥85 quadrats were sampled at ≥2 mooring buoys both within and outside marine protected areas, counts were summed to the transect level (Table 1). The resulting, replicate sums were scaled to numbers m^−2^.

To document the contribution of coral skeletons to the structure of reefs, coral colonies under each transect line were identified to the lowest possible taxonomic level, and the maximum diameters of their skeletons were measured. In 10 years when ≥50 colonies were sampled at ≥2 mooring buoys both within and outside marine protected areas, size frequency distributions were created by pooling data from transects surveyed around each mooring buoy (Table 1). The analyses focused on 26 species that represented the common corals in the region. Size classes ranged to ≥190 cm in 10-cm intervals, with the upper size class selected so that at least one species had 10 or more colonies in this bin. Counts per m for all size classes were scaled to a 100-m, linear search area to account for differing numbers of 10-m transects.

Live coral cover and numbers of juveniles m^−2^ were analyzed with a general linear model that treated year of sampling and degree of protection treated as orthogonal, fixed factors. Proportions for live coral cover were arcsin transformed prior to analysis, Type III sums of squares were used to account for unbalanced replication, and Cochran’s and Anderson–Darling tests were applied to residuals to evaluate compliance with the assumptions of homoscedasticity and normality, respectively.

Scaled counts for combinations of species and size classes were analyzed with a multivariate permutation analysis of variance (PERMANOVA) with years of sampling and levels of protection as orthogonal, fixed factors [30]. Thus, the PERMANOVA assessed significant differences among years of sampling and between levels of protection for both taxonomic composition and size frequency distributions of live coral assemblages, with the latter metric providing insights into differences or changes in the structure of the reef. A permutation analysis of multivariate dispersion (PERMDISP; [30]) was used to determine if any significant difference was due to increased variation among samples rather than a difference in the mean numbers of colonies in combinations of size classes and taxa. Finally, a similarities percentage routine (SIMPER; [30]) identified the primary combinations of size classes and taxa contributing to any significant difference detected by the PERMANOVA.

## Results

**Figure 2:**
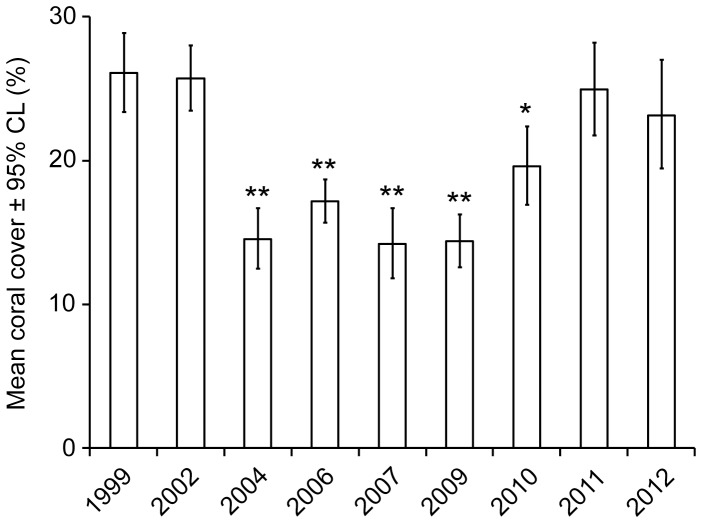
Back-transformed mean coral cover and 95% confidence limits (95% CL). Values based on replicate 10-m transects surveyed in 9–13 m of water during the years shown. ** = significantly different from 1999 at p ≤ 0.001; * = significantly different from 1999 at p ≤ 0.05; replicate transects surveyed in each year: 1999 = 61, 2002 = 50, 2004 = 47, 2006 = 41, 2007 = 52, 2009 = 34, 2010 = 52, 2011 = 29, 2012 = 30.

The proportional cover of live coral along line intercept transects exhibited statistically similar temporal trajectories in protected and unprotected areas (Table 2). Transformation did not yield homoscedasticity, but the results appeared reliable. The lack of a significant interaction and significant difference between protected and unprotected areas certainly represented robust results due to the unidirectional bias introduced by heteroscedasticity. The significant variation among years also appeared informative due to the level of significance and results of Dunnett’s multiple comparisons with 1999 treated as a control. Relative to 1999 levels, mean coral cover decreased significantly from 2002 to 2004 (26% to 14%), remained significantly lower through 2010, returned to a level that was not significantly different in 2011, and remained at that level in 2012 (Fig. 2). In both 2010 and 2011, mean cover of live coral increased by 5%. Thus, coral cover increased despite a localized bleaching event recorded in 2009 [28].

**Table 2:** Results of analysis of variance and tests of assumptions applied to arcsin transformed proportional live coral cover along transects and numbers of juveniles m^−2^.

Parameter	Anderson–Darling test	Cochran’s test	Source	df	SS	MS	F	p
Live coral cover	p = 0.02	p<0.01	Yr	8	1.361	0.170	14.29	<0.001
			MPA	1	0.032	0.032	2.72	0.100
			Yr × MPA	8	0.055	0.007	0.58	0.793
			Residual	378	4.500	0.012		
Juveniles m^−2^	p>0.05	p = 0.01	Yr	9	600.03	66.67	14.35	<0.001
			MPA	1	0.25	0.25	0.05	0.819
			Yr × MPA	9	49.68	5.52	1.19	0.322
			Residual	52	241.59	4.65		

Yr = year of survey, MPA = inside or outside of a marine protected area.

**Figure 3:**
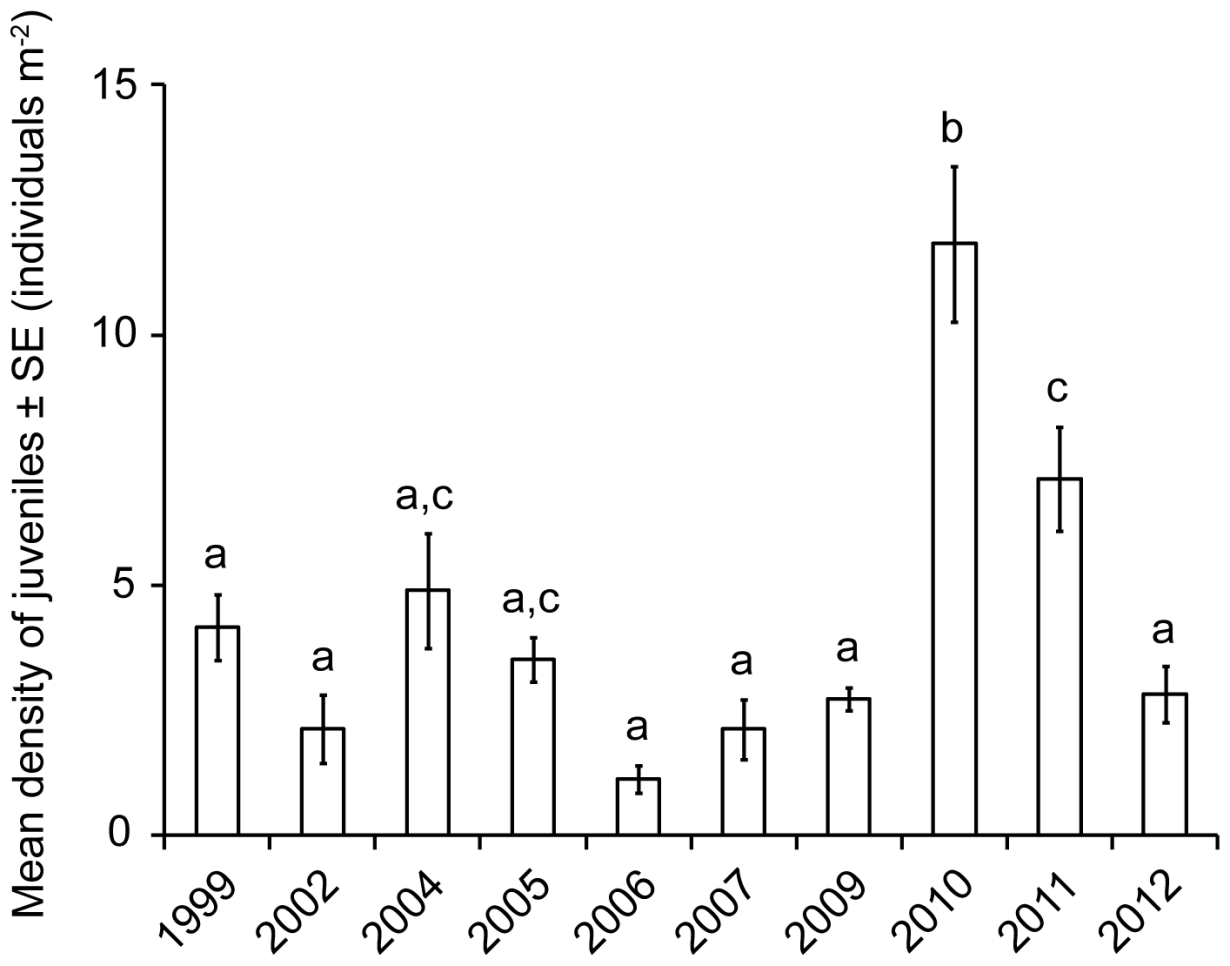
Mean numbers of juveniles m^−2^± standard errors (SE). Data recorded along replicate transects in the years shown. Means with different letters statistically different at p ≤ 0.05; replicate quadrats surveyed in each year: 1999 = 485, 2002 = 235, 2004 = 385, 205 = 235, 2006 = 315, 2007 = 345, 2009 = 255, 2010 = 180, 2011 = 235, 2012 = 300.

Densities of coral colonies ≤2 cm in diameter also exhibited statistically similar temporal trajectories in protected and unprotected areas (Table 2). According to Tukey’s tests, numbers of juveniles m^−2^ in 2010 were significantly higher than densities in all other years, and densities recorded in 2011 also were relatively high, albeit not statistically different from densities recorded in 2004 and 2005 (Fig. 3). Although both live coral cover and densities of juveniles increased contemporaneously, densities were not correlated significantly with coral cover in the following year (Pearson correlation coefficient = 0.689, p = 0.06).

In total, size classes for 26 species yielded 186 categories. The numbers of coral colonies in these categories differed significantly among years and between protected and unprotected areas (Table 3). Only the differences among years reflected increased variability among replicate samples as shown by the results for the permutation analyses of dispersion (PERMDISP; Table 4). Notably, coral assemblages displayed consistent temporal trajectories within and outside protected areas and consistent spatial patterns across years as evidenced by the nonsignificant interaction term (Table 3).

**Table 3:** Results of permutation analysis of variance applied to size classes of coral taxa scaled to numbers per 100-m, linear search area.

Source	Permutation analysis of variance					
	df	SS	MS	F	p	Unique permutations
Yr	9	38121	4236	2.30	0.001	993
MPA	1	5323	5323	2.89	0.003	998
Yr × MPA	9	13436	1493	0.81	0.954	997
Residual	51	123260	2417			

Yr = year of survey, MPA = inside or outside of a marine protected area.

**Table 4:** Results of permutation analysis of dispersion applied to size classes of coral taxa scaled to numbers per 100-m, linear search area.

Source	Permutation analysis of dispersion			
	df	F	p	Unique permutations
Yr	9, 61	2.91	0.045	999
MPA	1, 69	0.14	0.706	999

Yr = year of survey, MPA = inside or outside of a marine protected area.

**Figure 4:**
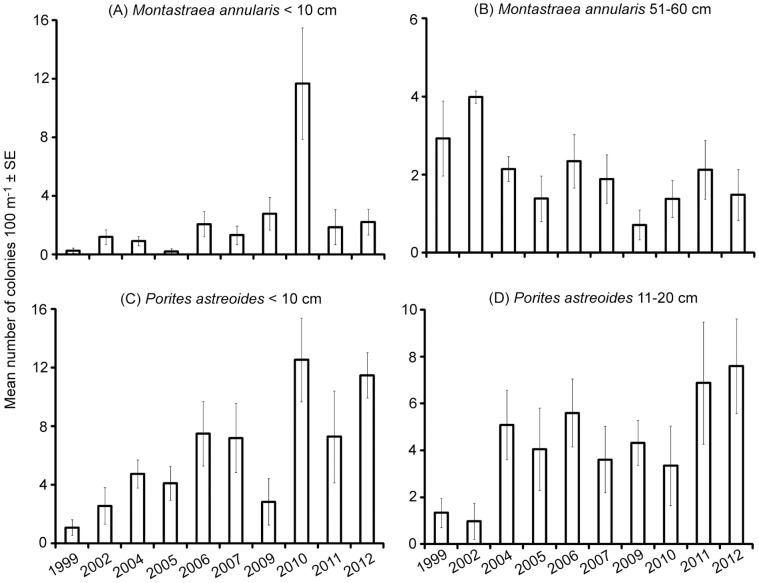
Mean numbers of coral colonies per 100-m, linear search area ± standard errors (SE). Means derived from ten years of sampling for (A) <10 cm *Montastraea annularis*, (B) 51–60 cm *M*. *annularis*, (C) <10 cm *Porites astreoides* and (D) 11–20 cm *P*. *astreoides*.

According to the SIMPER analysis for the 45 possible pairs of years, 41 to 79 combinations of size class and coral species accounted for 90% of the differences, with results for two size classes of *Montastraea annularis* and *Porites astreoides* illustrating key patterns (Fig. 4). Larger standard errors for means from some years illustrated the increased variability detected by the permutation analysis of dispersion (Table 4). Nevertheless, clear patterns were discernible. In 2010, relatively large numbers of <10 cm colonies were measured along transects for these two species (Fig. 4A and C) and for *Colpophyllia natans*, *Eusmilia fastigata*, *Madracis mirabilis*, *Manicina aerolata*, *Millepora alcicornis*, *M*. *cavernosa*, *M*. *faveolata*, *M*. *franksi*, *S*. *radians*, *Siderastrea siderea* and *Stephanocoenia intersepta*. For *M*. *annularis*, there tended to be fewer 51–60 cm colonies after 2002 and relatively stable numbers from 2004 to 2012 (Fig. 4B), which was a pattern observed for other size classes of *M*. *annularis* and some size classes of *Meandrina meandrites*, *M*. *cavernosa*, *M*. *faveolata* and *M*. *franksi*. In contrast, numbers of 11–20 cm *P*. *astreoides* did not decline consistently during the same years (Fig. 4C). Similarly, results indicated that numbers of colonies in regularly observed size classes of *Acropora palmata*, *C*. *natans*, *M*. *alcicornis*, and *S*. *siderea* did not decrease during this study. Furthermore, larger colonies (≥100 cm) of *Montastraea* species also persisted. Overall, the hard coral assemblages in protected and unprotected areas at Little Cayman Island remained relatively consistent, with 87% of the maximum differences among paired years being ≤2 colonies per 100-m, linear search area.

**Figure 5:**
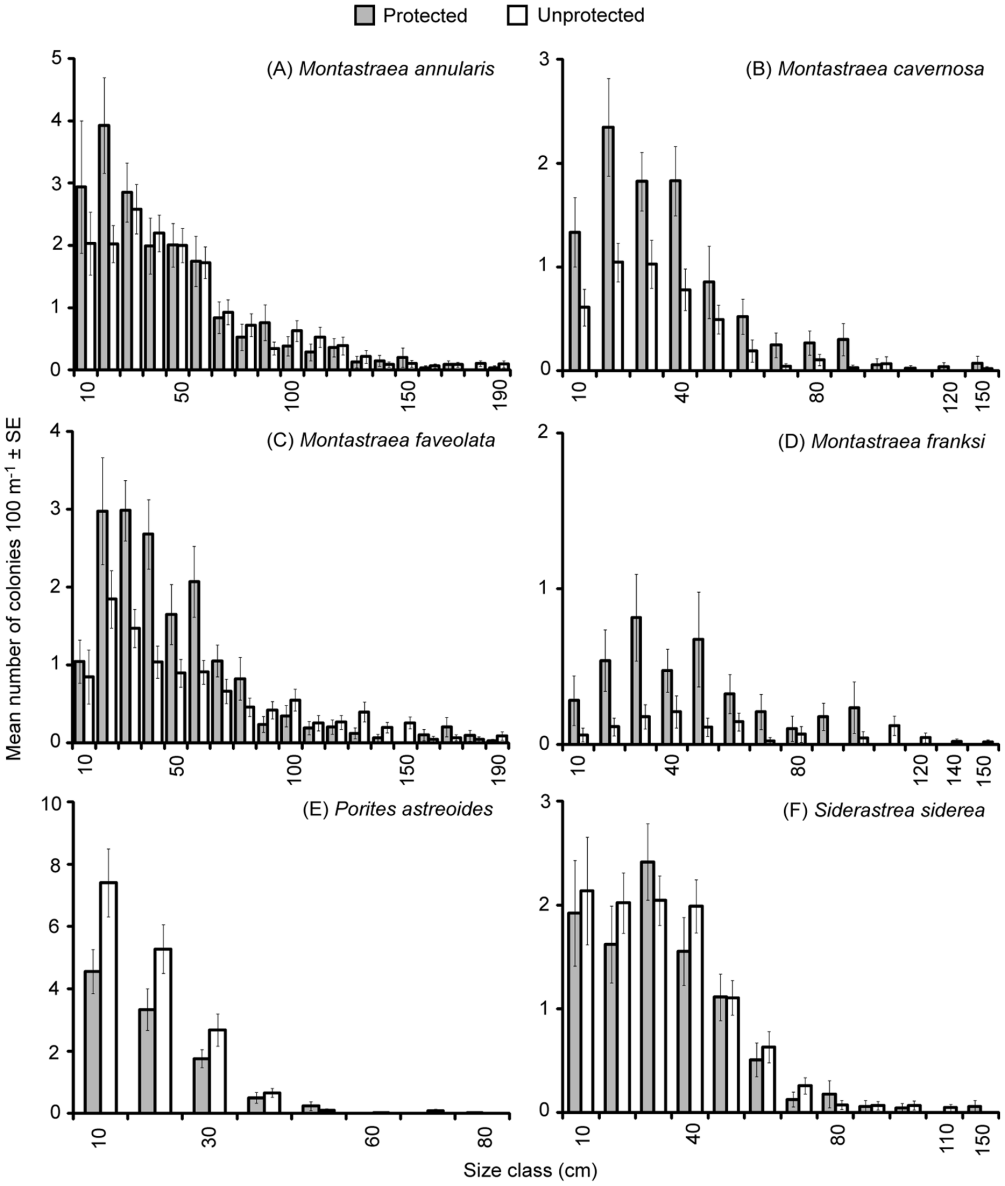
Mean numbers of colonies per 100-m, linear search area ± standard errors (SE). Means calculated for different size classes of (A) *Montastraea annularis*, (B) *M*. *cavernosa*, (C) *M. faveolata*, (D) *M*. *franksi*, (E) *Porites astreoides* and (F) *Siderastrea siderea* found within and outside marine protected areas. Note: some 10-cm size classes were omitted for clarity.

Out of the 186 combinations of size classes and coral taxa, a SIMPER analysis indicated that 75 accounted for 90% of the difference in the single comparison of assemblages within and outside marine protected areas. Numbers of colonies in different size classes for *M*. *annularis*, *M*. *cavernosa*, *M*. *faveolata*, *M*. *franksi*, *P*. *astreoides*, and *S*. *siderea* illustrated key patterns (Fig. 5). In this case, the consistency of standard errors reinforced the lack of a significant result for the permutation analysis of dispersion (Table 4). Means did vary between protected and unprotected areas, but the differences were <2 colonies per 100-m, linear search area (Fig. 5). *Montastraea* species, especially *M*. *cavernosa*, *M*. *faveolata* up to 80 cm in diameter and *M*. *franksi* up to 100 cm in diameter tended to be found in higher numbers within protected areas (Fig. 5A, B, C and D), whereas colonies of *P*. *astreoides* tended to be found in unprotected areas and *S*. *siderea* did not exhibit a consistent pattern (Fig. 5E and F).

## Discussion

Surveys spanning 14 years documented several ecologically significant results for coral assemblages off Little Cayman Island. Live coral cover decreased from 26% to 14% following thermal stress, bleaching and disease [9], [27], [28], but cover recovered to previous levels from 2010 onward. In 2010, significantly higher densities of colonies ≤2 cm in diameter provided evidence of a relatively large recruitment event. Minor changes in assemblage structure were detected via analyses of size classes of 26 species of coral, with decreases in some size classes documented after 2002 for species of *Montastraea* and *M*. *meandrites*, but not for other corals. In addition, only minor differences in assemblages within and outside marine protected areas were detected, with some size classes of some species more common within protected areas and others more common outside protected areas. Notably, temporal trajectories for all metrics did not differ significantly between locations within and outside marine protected areas, i.e., there were no significant interactions in the statistical analyses.

During recovery of live coral cover off Little Cayman Island, the increase from 14% to 20% between 2009 and 2010 and the increase from 20% to 25% between 2010 and 2011 were slightly less than the median increase in cover of 8% y^−1^ derived from previous reports of colony growth or colony recovery after a variety of disturbances on 67 reefs with <1% to 74% coral cover [13], [16], [17], [21], [31]–[33]. Thus, recovery of live coral cover at Little Cayman Island was on par with recoveries reported from other locations, and it occurred at the same rate within and outside protected areas.

In combination, an increase in densities of juvenile colonies and increases in small colonies of 13 coral species highlighted a relatively strong pulse of recruitment. Throughout the 14 years, reefs at Little Cayman Island received reasonable numbers of recruits (2–12 m^−2^). In fact, these densities of coral colonies ≤2 cm in diameter were i) higher than records from the Great Barrier Reef where colonies ≤2 cm in diameter represented juveniles (0.1–0.8 m^−2^; [12]); ii) similar to values from the Northern Line Islands where juveniles were designated as 1–5 cm colonies (1–10 m^−2^; [19]); iii) lower than maximum densities observed in Jamaica where juveniles were classified as 2–4 cm colonies (1–212 m^−2^; [34], [35]); and iv) lower than maximum densities observed in Curaçao, Bonaire, St. John in the U.S. Virgin Islands, the Florida Keys and Belize where colonies ≤5 cm were considered juveniles (1–44 m^−2^; [36]–[38]). Numbers of juveniles were not significantly correlated with increases in live coral cover during the following year, which suggested the need for further investigation to determine how variation in prevailing oceanographic conditions affects recruitment and how recruitment relates to recovery of live coral cover [17].

Overall, the size frequency distributions of hard coral assemblages in protected and unprotected areas at Little Cayman Island remained relatively consistent, with 87% of the maximum differences among paired years being ≤2 colonies per 100-m, linear search area. Numbers of larger colonies of *M*. *annularis*, *M*. *cavernosa*, *M*. *faveolata* and *M*. *franksi* and *M*. *meandrites* decreased in years following bleaching events and disease outbreaks [9], but numbers did not continue to decline as reported for these species at other Caribbean locations [39], [40]. In addition, numbers of *P*. *astreoides*, *A*. *palmata*, *C*. *natans*, *M*. *alcicornis*, and *S*. *siderea* did not decline consistently during the years surveyed, which suggested resistance to bleaching and disease [32], [41], [42]. At Little Cayman Island, *P*. *astreoides* and *S*. *siderea* were reported to suffer the lowest rates of bleaching and mortality [9], [28], but the same species, congeners and *A*. *palmata* have been reported to suffer bleaching and mortality elsewhere [42]–[49]. Perhaps, local oceanographic conditions, including adjacent deep water, ameliorated the effects of widespread thermal stress [9], [27], [28]. Colony size, physiological condition and genetic variation also influence resistance to stress, with greater tolerance documented for some coral species, larger colonies and populations exposed to repeated stress [41], [42]. In fact, some larger colonies of massive species, e.g., *Montastraea* species, persisted at Little Cayman Island. In contrast, the relative absence of local stressors at Little Cayman Island would not enhance resistance. Nevertheless, local adaptation by corals can be promoted if phylogeographical barriers reduce gene flow across a species’ range, as reported for Caribbean populations of *A*. *palmata* [50]. Further work is needed to disentangle the influences of genetics, physiology and environmental conditions on the persistence of coral colonies.

This study of coral reefs off Little Cayman Island demonstrated recovery after disease combined with regional thermal stress to cause a decrease in live coral cover from 26% to 14% over a five-year period. The temporal trajectories of the decline and subsequent recovery were similar within and outside protected areas, and the assemblage composition also remained similar across protected and unprotected sites throughout the decline and recovery. In addition, juvenile corals achieved similar densities within and outside protected areas. Key factors shaping recovery of corals off Little Cayman probably included the isolated geographic setting; stringent protection of a significant portion of the reefs resulting in healthy populations of herbivorous fishes and preservation of key trophic links; and minimal stress from local human activities.

Although any documented recovery of coral is encouraging, it is unlikely that such positive effects will spread throughout the Caribbean unless protection from local stresses is improved [51]–[53]. Without such improvements, recovery from natural cataclysms, including those exacerbated by global change, remains unlikely. Ultimately, management of local and global stresses will be required to sustain coral reefs and ensure their capacity to recover from disturbance [1].
